# Detection technologies and sensing systems for crop pest identification and infestation severity prediction: a review

**DOI:** 10.3389/fpls.2026.1765363

**Published:** 2026-02-27

**Authors:** Tianhua Chen, Yafei Wang, Jingmin Dang, Fu Zhang, Changji Wang

**Affiliations:** 1 College of Biological and Agricultural Engineering, Jilin University, Changchun, China; 2 College of Agricultural Equipment Engineering, Henan University of Science and Technology, Luoyang, China; 3 School of Agricultural Engineering, Jiangsu University, Zhenjiang, China; 4 Materials Science and Engineering, Henan University of Science and Technology, Luoyang, China

**Keywords:** infrared absorption spectroscopy, key information on crop physiological status, pest detection, trace gas sensors, volatile organic compounds (VOCs)

## Abstract

With the rapid development of precision agriculture technology, agricultural production is gradually shifting from traditional experience-based practices to data-driven decision-making. Pest species identification and scale prediction are crucial technologies in the field of pest detection. Compared with traditional pest monitoring methods, detection based on organic volatile gases released by crops under pest stress provides superior temporal and spatial resolution. The use of gas sensors in crop pest monitoring has great potential for application in future agricultural production. Infrared absorption spectroscopy-based gas sensors have gained widespread attention in crop pest monitoring due to their superior detection sensitivity and extensive scalability. A comprehensive overview of recent advances in intelligent detection methods and equipment for crop pest monitoring is provided. Emphasis is placed on the architecture, operating principles, sensing mechanisms, and fabrication materials of trace gas sensors based on infrared absorption spectroscopy for agricultural pest monitoring. In addition, key technologies involved in their fabrication processes are outlined. Finally, based on the specific characteristics of these sensors, the paper discusses in detail the application strategies of infrared absorption spectroscopy trace gas sensors in crop pest and disease monitoring, including transmission network design, platform integration, and the technical bottlenecks encountered in practical applications. The research will provide scientific foundations and innovative ideas for the development of future crop pest monitoring technologies, addressing the challenges faced by precision agriculture today.

## 1 Introduction

Ensuring global food security remains a critical challenge. It is projected that the global population will surge from 7.8 billion to 9.8 billion by 2050 (United Nations, 2019). As the primary food source for humanity, ensuring healthy crop growth and preventing shortages are therefore major priorities for global agriculture (Nelson et al., 2019). Pests can disrupt the normal growth and development of crops, adversely affecting their quality and yield, posing a significant threat to agricultural production (Cerda et al., 2017). Currently, in agricultural production, the blind overuse of chemical agents to combat pests not only increases agricultural costs but also causes numerous food safety and environmental security issues (Saik, 2024). Pests generally exhibit migratory and concealed behaviors, as well as self-protective habits. Pests can be classified into leaf-eating pests, stem-boring pests, piercing-sucking pests, and subterranean pests. Despite the diversity and notable achievements of existing monitoring technologies, their general applicability in complex and dense field crop environments remain limited. Previous pest monitoring research methods have problems such as limited spatial resolution, high cost, complicated operation, poor immediacy, inability to cope with foreign objects with the same spectrum, and static elements occlusion and other scenarios. Therefore, new methods are urgently needed to enable rapid pest identification, staging, and precise crop health assessment in the field. This will support efficient, targeted control measures, minimizing pesticide use and environmental impact, while meeting core agricultural needs from sowing to harvest.

To tackle this challenge, the remarkable sensory abilities of many organisms in nature have provided valuable inspiration for people. Many scholars have noticed that crops can coordinate and interact with the external environment by releasing organic volatile gases, which can serve as a key to exploring the physiological state information of crop and are important signals that cannot be ignored in understanding the survival status of crops (Kong, 2021; Raza et al., 2021; Sharma et al., 2023). In particular, the odor of crops after being subjected to pest stress is a significant indicator of the warning signal (Degenhardt, 2009). The interaction between plants and insects is one of the oldest interactions witnessed in nature since the emergence of both species. These two organisms constantly interact for their own benefit and have developed overwhelming strategies against each other (Indrakant et al., 2021). The release of organic volatile gases is an important means for plants to convey information and protect themselves (Li et al., 2021; Lin et al., 2022; Liu et al., 2023). When plants suffer herbivory (e.g., chewing, piercing), damage triggers hormonal responses. Key defense hormones like *jasmonic acid* activate specific gene expression. The resulting enzymes catalyze VOCs biosynthesis, and these volatile compounds are then released via stomata to serve ecological functions, such as attracting natural enemies or warning neighboring plants. The parasitoid wasps can precisely locate the location of pest infestations in a complex field environment based on the gases produced by plants after pest damage (Ortiz-Carreon et al., 2019; Smith, 1996). Moreover, the types and concentration of gases produced by different types, stages, and densities of pests vary (McCormick et al., 2014; Silva et al., 2017). Therefore, through *in-situ* flux detection and diagnosis of pest conditions in field crops based on volatile substances produced by plants, the types and scale of pest infestations can be predicted. Gas sensors are widely used in various fields due to their excellent flexibility, scalability, biocompatibility, fast detection speed, non-destructive sampling, simple operation, good stability, pollution-free nature, and low cost (Li et al., 2022; Mhanna et al., 2021). By targeting gases for detection, real-time, non-destructive, and continuous *in-situ* pest monitoring can be carried out through qualitative and quantitative analysis of the organic volatile gases produced by pest stress, helping to identify pest species and infestation stages, which is beneficial for implementing precision agriculture.

However, detecting pest-induced plant volatiles in dense field crops is challenging due to their low production rate and concentration, rapid dilution by air circulation, and the confounding background of diverse biological and microbial factors. Consequently, effective sensors require both ultra-low detection limits and high selectivity to identify target VOCs within complex gas mixtures (Ma et al., 2025). Infrared absorption spectroscopy has thus gained attention for trace gas sensing. It offers high sensitivity and selectivity, capable of resolving complex, overlapping spectral features to identify specific gases at parts-per-trillion levels. The main principle is to direct a beam of infrared rays onto the molecules of a substance, where specific wavelengths are absorbed, forming the infrared absorption spectrum of the molecule. Nearly all organic substances have detectable signals in the infrared spectrum, and their spectra are stable and easily accessible. For instance, in atmospheric monitoring, Krzempek et al. (2013) reported an optical multipass cell with an effective optical path length of 57.6 m, achieved by 459 reflections between two mirrors 12.5 cm apart, used to measure ethane concentrations, and ultimately achieving a minimum detection limit of 740 ppt with a 1s lock-in amplifier time constant. Liu et al. (2015) utilized a silver-coated concave spherical mirror and a 1.653 m fiber-coupled distributed feedback diode laser in a 280 cm^3^ optical cavity. They developed an optical multipass cell with an effective optical path length of 26.4 m, achieving 215 reflections and detecting methane in the atmosphere, with a final detection precision of 100 ppb. Compared to other types of gas sensors, the advantage of infrared absorption spectroscopy-based trace gas sensors lies in their ability to detect multiple gases simultaneously using a single light source sensor. The number of components used, system power consumption, volume, and cost are controllable, and the manufacturing process is simple. These sensors have demonstrated absolute advantages in detection accuracy. Additionally, with a wide range of mounting options, infrared absorption spectroscopy-based trace gas sensors can be mounted on aerial platforms or fixed or mobile ground platforms to carry out pest monitoring tasks, making them suitable for large-scale, dense crop monitoring.

**Figure 1:**
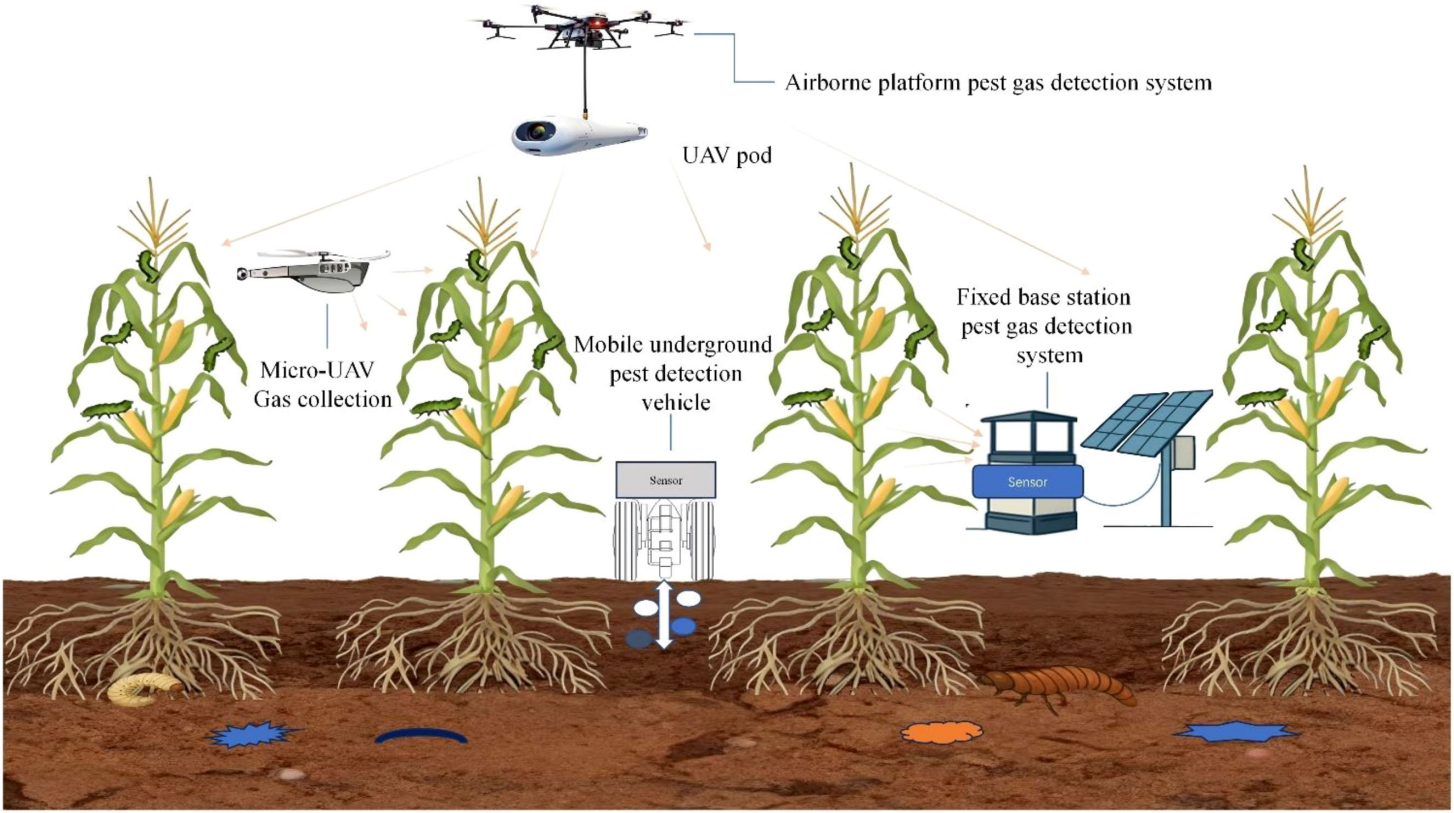
Schematic of multi-device integration and collaborative precise monitoring of crop pests.

With the shift towards data-driven agricultural management, gas sensors based on infrared absorption spectroscopy are valued for their high sensitivity and scalability. These sensors offer notable advantages in detecting faint gas signals under conditions of low pest density or early infestation. Pest species and infestation levels can be estimated both qualitatively and quantitatively using this technology. Supported by stable hardware and clear integration pathways, such sensors are highly suitable for real-time online monitoring in both field and facility agriculture. By enabling seamless compatibility with IoT platforms and sophisticated analytical algorithms, they form the backbone of precision pest monitoring and early warning systems that integrate multi-source data. This review systematically outlines recent advances in pest monitoring methods and gas-sensing technologies. It highlights the important role of gas sensing in precision agriculture for pest surveillance, with a particular focus on the structure, operating principles, and detection mechanisms of infrared absorption–based trace gas sensors. Current technical limitations are examined in light of practical application scenarios. Key challenges and future prospects for the use of such sensors in precision agricultural pest management are also discussed (**Figure 1**).

## 2 Typical pest detection methods

Conventional pest detection is chiefly reliant upon manual, periodic field surveys, predominantly point-based ground investigations, a method which is ill-suited to the demands of large-scale scientific monitoring and control. Agricultural production is gradually transforming from traditional experience to data-driven decision-making. The field of crop pest monitoring has witnessed substantial advancements in research, with the available technology now falling into two broad categories: direct image diagnostics and indirect biological information inversion techniques (He et al., 2016). The former includes airborne remote sensing technology and ground-based image recognition technology, which monitors physical differences in crop features such as leaves, fruits, and roots to determine the crop growth and development status. These technologies provide scientific data for agricultural production to some extent, but they still have limitations when used in complex field environments, such as low accuracy in pest detection and the inability to provide timely and accurate pest information for guiding agricultural production. Compared with the former, in indirect bioinformatic inversion technology, gas detection technology provides a new direction and new means for pest detection, which directly monitors the gases produced by crops after suffering from pest stress and utilizes the spectral characteristics of different gaseous substances as the detection results, which are processed and analyzed to invert the prediction of pest species and scale. This method offers higher spatiotemporal resolution for detecting immature and concealed pests, which are difficult to identify visually. **Table 1** Typical pest monitoring methods used to monitor intensively grown crops in agricultural fields.

**Table 1:** Typical pest monitoring methods of intensively grown crops.

Methods	Plant	Pest	Characteristics & Accuracy (%)	Ref
Remote sensing technology	Pines	*Southern pine beetle*	Overall accuracy increased from 84.7% to 90.1%	(Meng et al., 2022)
	Reed	*Locusta migratoria manilensish*	Unmanned aerial vehicle, RMSEs ranging from 8.8 to 9.1 g/m	(Song et al., 2020)
	Wheat	*Aphid/Schizaphis graminum/Mayetiola destructor*	Accuracy higher than 80%, Correlation coefficient ranged from 0.82 to0.98	(Bhattarai et al., 2019; Ma et al., 2019; Mirik et al., 2006)
	Capsicum annuum	*Two-spotted* sp*ider mites*	Acquire hyperspectral data using an integrating sphere to create vegetation indices for early Two-spotted spider mites’ detection	(Herrmann et al., 2012)
	Maize	*Armyworm/spider*	Accuracy increased from 0.50 to 0.79, The results support remote pest monitoring using reflectance	(Nansen et al., 2013; Zhang et al., 2015)
	Soybean	*Soybean aphid*	Accuracy of 89.4%/Sensitivity of 81.2%/Specificity of 91.6%	(Marston et al., 2022)
Image recognition technology	Rice	*Cnaphalocrcis medinalis/Chilo suppressalis*	Average precision of 90.11%	(Li et al., 2022)
	Cotton	*Spodoptera litura/Cotton bollworm/Cotton Aphid*	Accuracy rate of 0.94, a mean average precision (mAP) of 0.95, and frames per second (FPS) of 49.7	(Gao et al., 2024)
	Soybean seeds	*Riptortus pedestris*	Errors of less than 15% for $\mu_{a}$ and less than 10% for $\mu_{s}^{'}$	(Chen et al., 2024)
	Strawberry	*Thrips*	Percent error of less than 2.25%	(Ebrahimi et al., 2017)
	Kiwis	*Apolygus lucorum/Green leafhopper*	Average precision was 95.9%/Recall rate was 93.9%/Frames per second was 155	(Xiong et al., 2024)
	Maize	*Armyworm/Bollworm/Athetis Lepigone/Little Gecko/Yellow Tige/Holotrichia Oblita/Holotrichia Parallela/Anomala Corpulenta/Agriotes Fuscicollis Miwa*	MAP and mRecall by 6.3% and 4.61% over YOLOv3	(Lv et al., 2022)

### 2.1 Remote sensing monitoring methods

**Figure 2:**
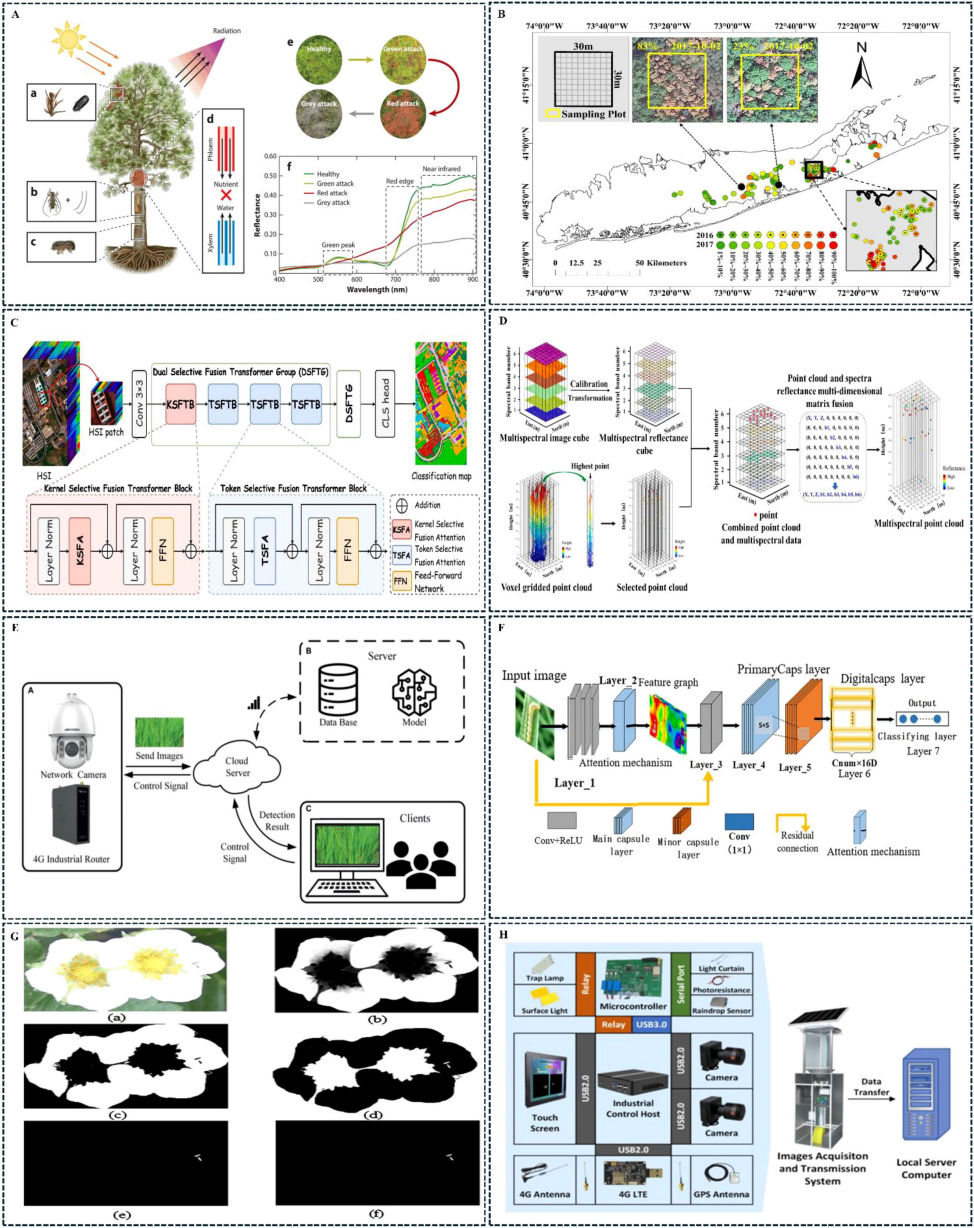
Applications of examples to identify typical crop pests. **(A)** (Luo et al., 2023);**(B)** (Meng et al., 2022); **(C)** (Xu et al., 2022); **(D)** (Gu et al., 2024); **(E)** (Li et al., 2022); **(F)** (Zhang et al., 2022); **(G)** (Ebrahimi et al., 2017); **(H)** (Liu et al., 2022).

Pests can cause changes in the physiological and structural characteristics of crops, causing the electromagnetic wave reflectance of crop leaves to change (Cao et al., 2022; Shi et al., 2018). Remote sensing technology has gained widespread recognition for its unique contribution in mapping the location, extent, and severity of crop pest outbreaks and assisting in analyzing potential factors that drive outbreak predictions (Xu et al., 2022). At present, remote sensing technology can acquire various crop information, including crop types, canopy temperature, physical characteristics, leaf nitrogen content, chemical composition, leaf inclination, structural traits, and crop density (Gu et al., 2024). Remote sensing has developed various systems for pest detection and monitoring, which utilize both active and passive radiation across a broad spectrum from gamma rays to microwaves. These include optical remote sensing, radar remote sensing, infrared remote sensing, as well as time-series and multi-temporal remote sensing. These technologies cover multiple bands, from visible light to infrared and microwaves, as well as different data formats and resolutions (Zheng et al., 2023). Luo et al. (2023) reported that after trees are attacked by wood-boring insects, the transport of water or nutrients may be interrupted, which affects the physiological state of the leaves and leads to changes in the spectral and structural information of the leaves and canopy. **Figure 2A** illustrates the remote sensing signal response to the biological and morphological changes caused by wood-boring pests. Meng et al. (2022) applied remote sensing technology to capture the regional scale of southern pine beetle infestation severity, enabling spatially explicit early warnings and detection of tree mortality caused by the beetle. Their results highlight the unique contribution of remote sensing in mapping the location, range, and intensity of beetle outbreaks, as well as in analyzing potential factors that drive outbreak predictions. **Figure 2B** illustrates the severity and spatial distribution of southern pine beetle damage after monitoring.

Researchers have explored remote sensing feature extraction, recognition, and classification algorithms at various scales. Notable methods include statistical discriminant analysis, machine learning, regression models, and spectral decomposition algorithms using single or multiple data collections. Xu et al. (2025) introduced a new dual-selective fusion transformer network for HSI classification to address the challenges of effective context scaling and ineffective self-attention features in context fusion when dealing with HSI scenes characterized by various land cover types and abundant spectral information. The study demonstrated that the proposed model significantly enhanced land cover classification accuracy, outperforming existing approaches. **Figure 2C** illustrates the data processing flow of the dual-selective fusion transformer network. Gu et al. (2024) employed motion structure combined with multi-view stereo algorithms to generate multispectral point clouds from drone-based multispectral images, which can also be used to estimate the 3D spatial distribution of crop photosynthetic parameters. Although the multispectral point clouds generated by structure from motion combined with multi-view stereo are sparse and incomplete, this method holds potential for application in the study of the 3D spatial distribution of crop parameters. **Figure 2D** shows a schematic of the fusion of lidar point clouds with multispectral images. However, when using remote sensing monitoring methods, certain limitations must be considered. In terms of scalability, remote sensing methods are well-suited for large-scale crop pest diagnosis and inspection, allowing for the identification of the area affected by pest damage. However, in practice, crop pest detection demands high resolution and precision from the equipment. Under complex climatic conditions, ordinary sensors cannot obtain accurate pest monitoring information in typical cloudy and foggy regions, and there are challenges in accurately identifying and distinguishing pest species in cases of overlapping spectra. For the future development of remote sensing technology, integrating multi-source remote sensing data for crop pest monitoring has already become a key direction for future research (Chen et al., 2025).

### 2.2 Image detection methods

The crop pest image recognition method mainly utilizes high-definition cameras and visual sensors to create image data collection terminals, which are mounted on fixed or movable platforms for capturing images of crops in targeted areas. After image acquisition, the data are transmitted to local or cloud servers equipped with algorithms for pest feature extraction and matching, enabling comprehensive analyses of pest species, population density, and development trends. **Figure 2E** shows the structure diagram of an intelligent rice canopy pest and disease monitoring system reported by (Li et al., 2022). The typical process for crop image recognition and detection includes image acquisition, image preprocessing, image segmentation, feature extraction, and classification (Lv et al., 2022). Zhang et al. (2022) attempted to solve the problem of noticeable differences in the same pest species under different shapes, colors, sizes, and complex backgrounds, by proposing a crop pest recognition method based on an improved capsule network (MCapsNet). In MCapsNet, capsule networks are used to enhance traditional convolutional neural networks, with an attention module introduced to capture the most critical classification features and speed up network training. **Figure 2F** shows the architecture of MCapsNet.

The performance of pest image recognition algorithms is crucial for pest detection applications. Machine learning has been widely applied to detect field crop pest images, and research on the detection algorithm architectures in existing image recognition methods mainly centers on support vector machine (SVM), convolutional neural network (CNN), and Transformer architectures. Ebrahimi et al. (2017) developed an automatic pest detection method for thrips in strawberry greenhouses based on a support vector machine classification approach, mainly utilizing the ratio of large to small diameters as a regional indicator and hue and saturation as color indicators to design the SVM structure. Experimental results indicated that the average error in pest recognition detection was less than 2.25%, and the segmentation results for thrips detection are shown in **Figure 2G**. SVM performs well with linearly separable data, but it may not be as effective as CNN in handling non-linear data. CNN can effectively capture local features in images and gradually build more complex and abstract feature representations. This ability makes CNN particularly effective in pest detection and recognition tasks. By stacking deep convolutional layers, CNN is the most effective method for automatically learning and extracting useful information from pest images, thereby enabling high-performance pest detection and recognition (Cheng et al., 2017). The most used algorithm at present is the CNN-based improvement of You Only Look Once (YOLO). The YOLO framework treats object detection as a regression problem, solving it with a single forward pass through the convolutional neural network. YOLO performs both object localization and recognition simultaneously across the entire image (Teng et al., 2022). By adopting global feature representations, multi-scale predictions, and dividing the input image into fixed grids, the YOLO series incorporates several enhancements during its iterative process to improve performance (Popescu et al., 2023). Xiong et al. (2024) focused on the green stink bug and small green leafhopper on kiwi and applied an improved YOLOv5s image recognition algorithm at high, medium, and low-density conditions. The results achieved a map of 96.3% and recall of 94.0%. The transformer model can analyses the temporal changes in pest behavior, offering powerful support for dynamic monitoring and early warning of pest activities. Gao et al. (2024) developed an intelligent detection model using transformer deep learning for fast detection of cotton pests and diseases. Experimental results indicated an accuracy of 0.94, a mean average precision f 0.95, and a frame rate of 49.7. Liu et al. (2022) combined deep learning algorithms to design a fixed light-induced insect image collection device to recognize and predict field pest conditions. Based on the ResNet V2 model, they trained T_ResNet V2 using a migration learning dataset. The recognition accuracy on the test set was 84.6%, and after reasonably combining indoor and field datasets, the recognition accuracy of the SM_ResNet V2 model reached 85.7%. The structure of the device is shown in **Figure 2H**. Despite considerable progress in crop pest image recognition methods for pest detection, which demonstrate their efficiency in practical applications, challenges arise in real agricultural environments. Variable lighting conditions, crop diversity and changes in the size, color and shape of pests at different stages make it difficult to accurately and robustly recognize pests in images, significantly increasing the complexity of identification and detection. Notably, there is limited research on existing image recognition methods for underground and concealed pests. Moreover, when detecting small pests with similar modes in the same spatiotemporal context, the dense distribution and small size of these pests call for further research and development of detection devices and algorithms to improve image recognition accuracy. These are key bottlenecks and challenges that need to be addressed to enhance pest detection precision.

## 3 Common gas sensors for crop monitoring

In nature, the ability of plants and animals to perceive odors is many times more sensitive than that of humans. Odors serve as a vital medium for information transfer and communication between plants and animals in nature. Researchers are progressively decoding the role of gases in this process, driving the development and application of numerous gas sensors. Sensors for crop status monitoring, characterized by light weight, miniaturization, intelligence, low cost, and portability, have become a key demand at present. Currently, commonly used gas sensors for plant health monitoring include types such as gas chromatography, electrochemical, semiconductor, and infrared absorption spectroscopy. Each type of gas sensor has a different range of applications. The subsequent discourse delineates the features of different gas sensors of plant gas sensors, as exhibited in **Table 2**.

**Table 2:** Features of different gas sensors.

Sensors	Detection limit	Selectivity	Response time	Cost	Anti-interference	Feasibility of field	Ref
Gas chromatographic	ppb	high	minute	expensive	high	requires professional operation	(Ranger et al., 2010; Weis et al., 2016)
Electrochemical	ppb to ppm	moderate	seconds	economical to medium	moderate	compact and portable	(Bulemo et al., 2025; Ibrahim et al., 2022)
Semiconductor	ppm	poor	seconds to minutes	economical	poor	compact and portable	(Li et al., 2023; Xu et al., 2025)
Infrared absorption spectroscopy	ppb to ppm	high	seconds to ten seconds	medium	relatively high	portable deployment feasible	(Zheng et al., 2023)

### 3.1 Gas chromatographic sensors

Gas chromatography gas sensors operate based on the differential distribution and adsorption of substances. Inert gases such as hydrogen, nitrogen, and helium are used as the mobile phase to separate and quantitatively measure different gases, essentially combining the high separation efficiency of the chromatographic column with high-sensitivity detection technology. While it provides accurate and objective results, its development and application have been restricted by challenges such as high detection costs, time consumption, complicated analytical procedures, and sample destruction (Weis et al., 2016). Ranger et al. (2010) used gas chromatography and gas sensors to verify the attraction of volatiles produced by nursery plants to ambrosia beetles and to demonstrate the ability of these beetles to attack selected trees when they are attracted by these volatiles. Gas chromatography gas sensors have evolved into several types, including GC-IMS, GC-O, GC × GC-MS, MS/GC × GC-MS/O, and GC-MS, with notable achievements in fields such as tea aroma detection. The miniaturization of instruments, portability, user-friendly human-machine interaction, and the ability to be mounted are crucial products urgently needed in pest detection. In the miniaturization of instruments, gas chromatographic sensors have developed microchip capillary electrophoresis gas chromatography (MCECGC). This technique uses the principle of capillary electrophoresis, where the electric field drives different sized charges to move and separate the gas components into the gas chromatographic part with a coated column, allowing for qualitative and quantitative analysis of the components. Compared to traditional capillary electrophoresis equipment, this is a sophisticated technique for analyzing complex mixtures, capable of achieving rapid separation and high resolution with minimal sample volumes. These miniature detection methods are still in their pregnancy, with only a few applications having been discussed (Nam et al., 2021; Wang et al., 2024; Wu et al., 2024).

### 3.2 Electrochemical gas sensor

**Figure 3:**
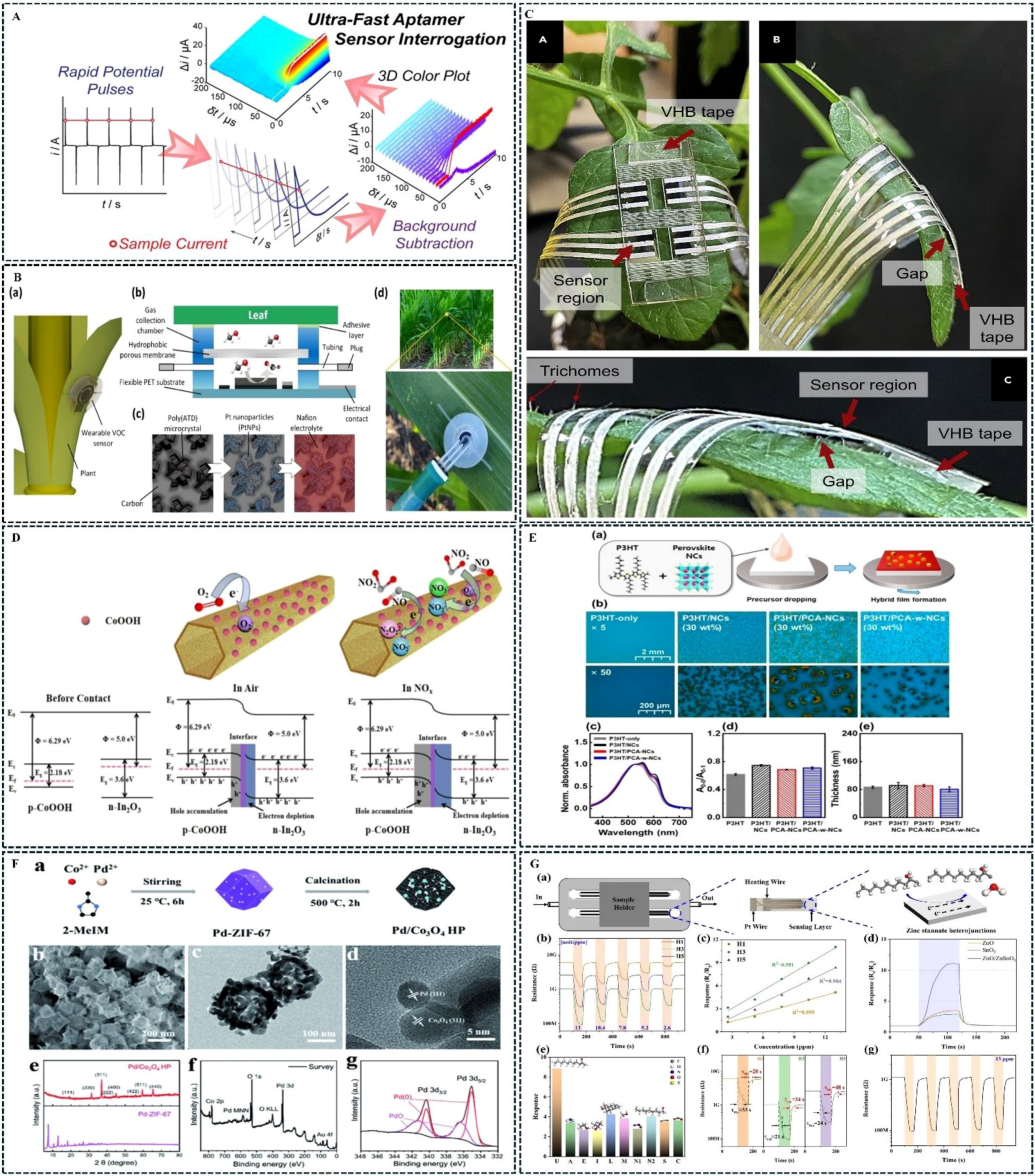
Application examples of common gas sensors. **(A)** (Mirelis et al., 2018); **(B)** (Ibrahim et al., 2022); **(C)** (Li et al., 2021); **(D)** (Liu et al., 2025); **(E)** (Jang et al., 2023); **(F)** (Yuan et al., 2022); **(G)** (Xu et al., 2025).

Electrochemical gas sensors detect gases through electrochemical reactions occurring between the target gas and the working electrode and reference electrode. Electrochemical sensor detection methods are renowned for their sensitivity and versatility, enabling real-time and on-site analysis with minimal sample volume. A typical sensor consists of a permeable membrane, electrodes, an electrolyte, filters, and a housing. This technique involves detecting the presence of analytes in the sample through changes in voltage, current, or resistance (Bulemo et al., 2025). In gas detection applications, gases enter the sensor and undergo oxidation or reduction reactions with the electrolyte or electrode surface, producing signals such as current, which are proportional to the analyte concentration (Patra et al., 2022). By measuring the signal strength, the gas concentration can be derived, and it has broad applications in fields such as food safety, environmental protection, and aerospace. As the main electrochemical sensing techniques, voltammetry and amperometry have distinct features. Amperometry performs well in continuous monitoring of specific substances, while voltammetry is particularly effective in analyzing detailed sample information (Shimali et al., 2024). Menart et al. (2017) developed an electrochemical gas sensor for formaldehyde gas. The sensor mainly consists of a tri-electrode mesh modified with polyacrylic hydrazine and a poly-electrolyte capable of voltametric measurements. Experimental results showed that the system exhibited linear response over a formaldehyde concentration range of 4–16 ppm under a 120-min accumulation time, with a determination coefficient (R²) of 0.976. Mirelis et al. (2018) employed intermittent pulse amperometry technology to investigate the equilibrium and kinetic binding of target analytes on the surface of electrochemical aptamer sensors. By applying potential pulses using IPA and detecting changes in charge transfer rate within<100μs, they confirmed that changes in sensor current were quantitatively correlated with the target analyte concentration. The intermittent pulse amperometry method offers unprecedented sub-microsecond time response and serves as a general approach for evaluating fast sensor performance, ultimately achieving time resolution up to 2 Ms. **Figure 3A** shows a schematic representation of the response structure of the aptamer-based electrochemical sensor using rapid, two-millisecond interrogation with intermittent pulse amperometry. Although electrochemical detection technologies face challenges such as limited sensor lifespan, high sensitivity to temperature requiring internal compensation for stability, high maintenance demands, and the volatility and leakage of liquid electrolytes affecting the sensor’s lifespan, the replacement of liquid electrolytes with solid-state and organic gel electrolytes has become the future development direction for electrochemical sensors (Williams, 2020).

The electrochemical sensor-based flexible wearable gas sensor, developed for monitoring plant physiological states, can continuously detect the organic volatile gases emitted by plants *in situ*, reflecting crucial physiological and pathological parameters. Additionally, the flexible sensor exhibits outstanding mechanical flexibility and strong adhesion to the non-flat surfaces of biological materials, providing a unique potential for real-time, *in-situ* monitoring of crop growth. Ibrahim et al. (2022) developed an electrochemical, wearable plant sensor for the *in-situ* monitoring of methanol emissions from plant leaves. This sensor uses platinum nanoparticles modified with the conductive polymer poly(2-amino-1,3,4-thiadiazole) as an active catalyst for the electrochemical oxidation of methanol at specific potentials. The sensor is integrated into a miniature gas collection chamber which is mounted on the leaf surface and covered with a hydrophobic gas diffusion membrane. It offers a detection range of 0.5–500 ppm and a response time of under 20 seconds, **Figure 3B** shows a schematic representation of the structure of a wearable electrochemical VOCs sensor for monitoring methanol emissions from maize. Li et al. (2021) presented an electrochemical gas sensor array that can be attached to leaves. The sensor patches are applied to a stretchable substrate and incorporate a variety of graphene-based sensing materials and flexible silver nanowire electrodes. This sensor can detect VOCs gas fingerprints generated by stressed crops in real time, accurately detecting and classifying 13 types of plant volatile with over 97% accuracy. **Figure 3C** shows a flexible sensor mounted on the tomato leaf. Despite challenges remaining in terms of material costs, wireless power, networking transmission and the destructive impact of pests such as stemborers and leaf-eating insects, remarkable progress has already been made in plant growth monitoring using flexible gas sensors. Plant wearable devices thus hold great potential for simple, accurate, and continuous large-scale health monitoring (Yan et al., 2024).

### 3.3 Semiconductor gas sensor

Semiconductor gas sensors detect gases based on the chemical adsorption properties of metal oxide semiconductors. Semiconductor materials are typically divided into n-type semiconductors, including SnO_2_, ZnO, TiO_2_, WO_3_, In_2_O_3_, Fe_2_O_3_, and CeO_2_, and p-type semiconductors, such as CuO, NiO, Co_3_O_4_, Cr_2_O_3_, and Mn_3_O_4_. When the target gas reacts with the semiconductor material on the sensor surface, conductivity changes are used to detect the gas. The key lies in the interaction between the gas and the material’s surface, involving theories such as the chemical adsorption oxygen model, grain boundary barrier model, bulk resistance model, and space charge layer model (Li et al., 2023). The detection principle is based on n-type semiconductor materials, where electrons act as electron donors. The electrons from the conduction band move towards the electrode, generating holes that are captured by the electron donors, completing the electrode reaction, thus increasing conductivity and forming an anodic photocurrent. In contrast, for p-type semiconductors, holes act as electron acceptors. Electrons on the electrode neutralize the holes in the valence band, lowering the conductivity and forming a cathodic photocurrent. These changes reflect the gas concentration by altering physical quantities like conductivity.

Co_3_O_4_, In_2_O_3_/CoOOH composites, as well as perovskite nanocrystals and conductive polymer blends, have gained widespread attention as novel gas-sensitive materials, demonstrating excellent stability and performance in detecting analytes such as H_2_, CO, NO, NH_3_, and VOCs. Koga (2020) employed pulsed laser ablation to successfully prepare Co_3_O_4_-NP aggregates with uniformly dispersed Pd additives. Their research revealed that at 5% Pd loading, the material achieved optimal H_2_ sensing performance with the highest density of single Pd atoms, which enhanced oxygen adsorption, free electron density, and the formation of high-concentration adsorbed ions. Furthermore, the excellent electron mobility and large surface area of these materials make them ideal for building gas sensors, solving problems such as high working temperatures, low sensitivity, and poor moisture resistance in single metal oxide materials. Liu et al. (2025) enhanced the gas sensing performance by constructing p-n heterostructures, which aid in the redistribution of electrons, improving the material’s response speed and sensitivity. Initially, sodium hydroxide (NaOH) solution was used to etch the synthesized metal-organic framework (MOF)-derived In_2_O_3_ nanotubes and Co-MOF precursors, leading to the preparation of MOF-derived In_2_O_3_/CoOOH composites. The structure and morphology of the composite Finally, *in situ* infrared spectroscopy was employed to study the NOx interaction in In_2_O_3_/CoOOH composites. At room temperature, the response value (Rg/Ra) for 10 ppm NOx was 84%, with a detection limit of 0.1 ppm, approximately 2.5 times that of pure In_2_O_3_. The response and recovery times were 141/78s, which is faster than the 179/86s for pure In_2_O_3_. material was then analyzed using techniques such as XRD, FT-IR, SEM, TEM, and XPS. **Figure 3D** shows the simulated sensing mechanism and energy bands of the In_2_O_3_/CoOOH composite material before and after exposure to nitrogen oxides. Jang et al. (2023) developed a novel organic-inorganic hybrid gas sensor by blending perovskite nanocrystals with conductive polymers to improve gas absorption capacity and overcome the challenges of low sensitivity and poor stability in organic semiconductors. CsPbBr_3_ perovskite was embedded in the conductive polymer matrix to enhance gas sensing performance while maintaining sensing speed, and amphoteric ion polymer ligands were used to modify the surface of the perovskite nanocrystals. The lowest detection limits were compared in the PCA-w-NCs system, with NO_2_ detection limit of 0.000318 ppb, SO_2_ at 0.00112 ppb, and CO_2_ at 0.00479 ppb. **Figure 3E** shows a schematic representation of the preparation method for the P_3_HT/perovskite blended film. Yuan et al. (2022) developed Pd-modified Co_3_O_4_ hollow polyhedral (Pd/Co_3_O_4_-HP) by pyrolyzing a Pd-doped MOF precursor and designed an ethanol gas sensor. The experiments demonstrated that at a working temperature of 150°C, the Pd/Co_3_O_4_-HP gas sensor achieved 1.6 times higher sensitivity compared to Co_3_O_4_-HP, with rapid response (12 seconds) and recovery (25 seconds) to 100 ppm ethanol vapor. This study provides an effective approach for investigating the spillover effect in the sensing mechanisms of precious metal-modified oxide semiconductor sensors. The preparation and characterization of Pd/Co_3_O_4_-HP are depicted in **Figure 3F**.

The electronic nose, based on semiconductor sensor technology, is an instrument that consists of an array of electronic gas sensors with partial specificity and pattern recognition algorithms. This gas sensor array technology is also termed an electronic nose. Inspired by the human olfactory system, during detection, the target gas is collected into a chamber with an array of electronic chemical gas sensors with partial specificity via a sampling pump. Different gas-sensitive sensors react specifically to various organic compounds, causing changes in the sensor’s resistance. The sensor reaches a stable state within a few seconds. The data acquisition system records real-time changes in the response signals from each sensor. After processing with suitable pattern recognition algorithms, the type and concentration of the detected gas are determined, enabling the identification of both simple and complex gases. Xu et al. (2025) leveraging the enhanced adsorption capability of semiconductors and dual mesoporous characteristics, developed an innovative method for fabricating zinc tin oxide semiconductors with dual mesoporous structures and tunable oxygen vacancies using direct solution precursor plasma spraying technology. This method was employed for detecting 2-undecanone, a key biomarker for rice aging. Experimental results showed that the developed sensor effectively detected adulteration in different rice varieties. The sensor’s sensitivity to VOCs from japonica rice was as high as 62.9%, about 4.5 times that of indica rice. The results of testing the gas-sensing properties of all materials on the platform of a side-heating gas sensor are shown in **Figure 3G**. Zhou (2011) focused on common pests in rice cultivation, such as the striped stem borer and brown planthopper. They used the developed electronic nose to detect organic volatile gases produced by rice plants under pest stress, compared with healthy rice plants. The experiment ultimately verified the feasibility of using the electronic nose for pest detection in rice plants. Despite the potential of the electronic nose in various fields, its application for pest detection in complex field environments still faces challenges. First, environmental factors such as temperature and humidity may affect sensor sensitivity. Interference from multiple coexisting gases leads to inaccurate identification, necessitating the development of new materials and technologies adapted to varying temperature environments. Secondly, sensors have poor long-term stability, requiring regular calibration or replacement. The cost and limited lifespan of high-performance sensors also add to the maintenance burden, while complex data processing demands significant computational resources. This limitation restricts the widespread use of electronic noses in high-precision applications, necessitating urgent solutions.

## 4 Infrared absorption spectroscopy trace gas sensors

Typical trace gas sensor detection system using infrared absorption spectroscopy usually includes a light source for detection, a discrete optical multi-pass cell, a detector and associated electrical modules. In practical applications, light passes through the gas being detected and the detector measures any change in light intensity. This process converts the optical signal into an electrical output, which is then used by devices such as acquisition cards to determine the gas concentration. Compared to other types of gas sensor, infrared absorption spectroscopy trace gas sensors have the advantage of being able to detect multiple gases simultaneously using a single light source sensor. The number of components used, system power consumption, volume and cost can all be controlled, and the manufacturing process is simple. They also demonstrate superior detection accuracy. Furthermore, they offer versatile deployment options and can be mounted on airborne or fixed/mobile ground platforms for pest monitoring tasks. The infrared absorption spectroscopy trace gas sensor is ideal for monitoring large areas of dense crops. According to the theory of molecular spectroscopy, when an infrared beam is absorbed by gas molecules, the different molecules in the gas sample absorb light at different frequencies. The intensity of the light decreases due to absorption by the gas molecules, and the degree of attenuation depends on factors such as the absorption coefficient, the concentration of the gas molecules, and the effective optical path length. Researchers have conducted extensive studies and achieved significant progress in improving the performance of lasers, optical multi-pass cells, and detectors to enhance the accuracy. The simultaneous detection of multiple gases, the development of long optical path multi-pass cells with limited volume, and the enhancement of high-precision detector performance have become the primary research focus in the development of infrared absorption spectroscopy trace gas sensors.

### 4.1 Lasers for simultaneous multi-gas detection

**Figure 4:**
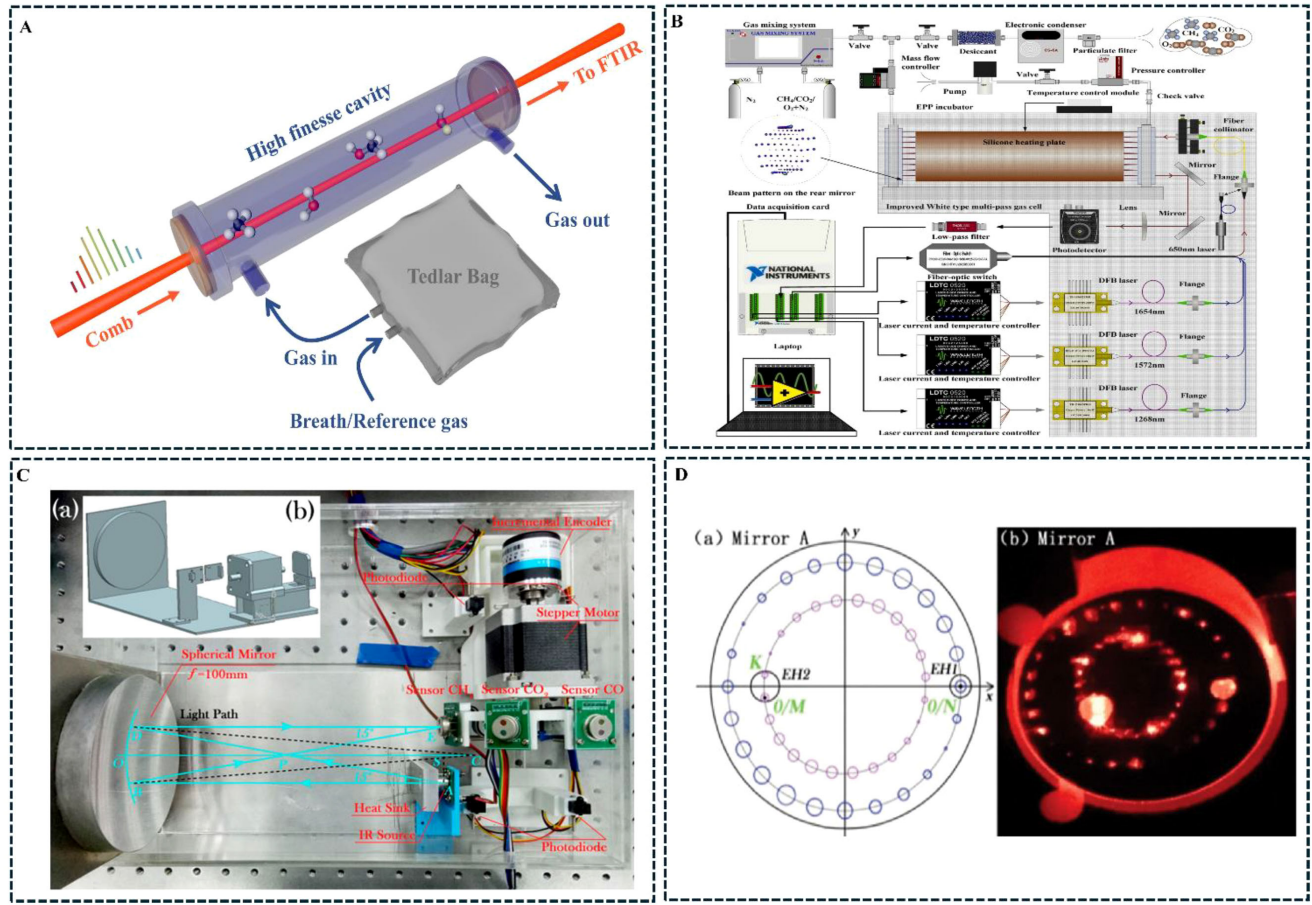
Application examples of multi-gas detection lasers. **(A)** (Liang et al., 2021); **(B)** (Dang et al., 2024); **(C)** (Dong et al., 2017); **(D)** (Dong et al., 2019).

The light source, as the core component of trace gas sensors in infrared absorption spectroscopy, should be selected based on the operational scenario, determining the spectral range of the target gas. The light source must have sufficiently high absorption intensity for the gas, be tunable within the wavelength range, avoid interference from other gas absorption lines, and match the selected spectral technique. Sources emitting infrared, visible, and ultraviolet spectral wavelengths can be selected. Furthermore, it’s important to note that different light sources have different radiation bandwidths. Lasers generate narrowband radiation, whereas thermally infrared coherent light sources generate broadband radiation. These distinct characteristics dictate the choice of spectroscopic technique: tunable laser absorption spectroscopy (TLAS) for narrowband laser sources, and non-dispersive infrared (NDIR) spectroscopy for broadband thermal sources. The choice of light source determines the type of optical multi-pass cell and detector used in the system, which indirectly affects the gas sensing system’s overall detection accuracy. The use of a single light source to detect multiple gases simultaneously in infrared absorption spectroscopy trace gas sensors has attracted significant interest from many researchers. In single light source systems, mid-infrared frequency comb spectroscopy technology has attracted attention for its outstanding ability to detect multiple gases. The frequency comb laser plays a critical role in this sensing technique by combining high spectral resolution, broad bandwidth coverage, and high-precision cavity interactions through individual comb teeth. Unlike conventional lasers, they can emit light pulses with thousands to millions of different “frequencies” that are coupled into the cavity. These pulses interact with molecules in the cavity as they make thousands of round trips in the gas pool, allowing for gas species identification (Liang et al., 2025). Liang et al. (2021) addressed the issue of resonance mode mismatches between mid-infrared light sources and optical cavities, which previously led to laser beam emission. They used the modulated ring-decreasing frequency comb interference method and employed mid-infrared cavity-enhanced frequency comb spectroscopy technology for human breath gas detection. Their research enabled the simultaneous detection and monitoring of four breath biomarkers—CH_3_OH, CH_4_, H_2_O, and HDO—with detection accuracy reaching ultra-high sensitivity at the trillionth level. The schematic diagram of the mid-infrared frequency comb detection process is shown in **Figure 4A**. Ben et al. (2016) applied infrared frequency comb spectroscopy technology to detect complex molecules of various gases. The study demonstrated a significant increase in spectral resolution and specificity in identifying multiple gas molecules.

The optical multiplexing structure of the light source is important for the simultaneous detection of multiple gases. It passes infrared light from different lasers through an optical multi-pass cell in sequence, enabling multiple gases to be detected simultaneously. Our team has developed a wavelength modulation spectroscopy technique that combines second harmonic detection and a laser power fluctuation correction method. Based on a fiber optic switch integrated with time-division multiplexing technology, this near-infrared distributed feedback long-path absorption spectroscopy laser sensor enables different lasers to sequentially scan and measure methane (CH_4_), carbon dioxide (CO_2_), and oxygen (O_2_) in the atmosphere. Experimental results indicate that, depending on concentration and noise levels, the detection limit (1σ) for CH_4_ can reach 0.034 ppm, for CO_2_ it can reach 11.921 ppm, and for O_2_ it can reach 0.14% (Dang et al., 2024). The multi-channel multiplexed multi-gas sensor based on the fiber optic switch is shown in **Figure 4B**. In another study, Dong et al. (2017) employed a single broadband light source and a fast-switching detector device to detect multiple gases. The method primarily used a stepper motor to rotate and switch between thermoelectric detectors for carbon monoxide (CO), carbon dioxide (CO_2_), and methane (CH_4_). The study demonstrated that the stability of the multi-gas sensor was optimal when the switching motor operated at a speed of 0.4π rad/s, achieving a detection limit of 8.83, 8.69, and 10.29 ppm for the three gases in one second of dynamic operation. The multi-detector rotating switch device is shown in **Figure 4C**. Furthermore, regarding the optical aperture for gas cell light passage, Dong et al. (2019) proposed a concentric reflection model design method to address the spatial waste issue in classic Herriott cells and improve mirror utilization efficiency. They developed a dual-ring structure optical multi-pass cell that implements multi-gas detection, enabling a single Herriott optical multi-pass cell to support multiple light paths and facilitate the simultaneous detection of either single or multiple gases. The optical multi-pass cell structure with two incident apertures is shown in **Figure 4D**.

### 4.2 Finite volume long optical path multi-pass cell

An optical multi-pass cell is designed to increase the effective optical path length for gas absorption. This is achieved by coating the mirror surface with various high-reflection materials, satisfying infrared absorption spectroscopy detection requirements across a range of wavelengths. Typical optical multi-pass absorption cell structures include White, Herriott, ring-type, and discrete lens long optical path cells. According to Lambert-Beer’s law, the strength of absorption is directly proportional to the effective interaction length between the laser and the gas. For gas molecules to absorb enough light, the light must travel several miles or even further through the gas sample in an optical cavity that is measured in feet. The length of the optical path dictates the detection accuracy of the gas sensor, so enhancing the effective absorption path length is a critical method for improving the detection sensitivity of the system. Precise cavity alignment is challenging, as mismatches between the laser and cavity resonant modes cause the beam to be rejected. Enhancing the efficiency of mirror utilization and reducing the volume of optical multi-pass cells are currently key areas of focus in research. Many researchers have developed various high-precision finite volume long optical path multi-pass cells tailored to different application scenarios, considering factors like light source type, light coupling methods, optical path structure, detection techniques, and target gases. New optical-mechanical structures are emerging, and optical multi-pass cells still have vast potential for improvement.

**Figure 5:**
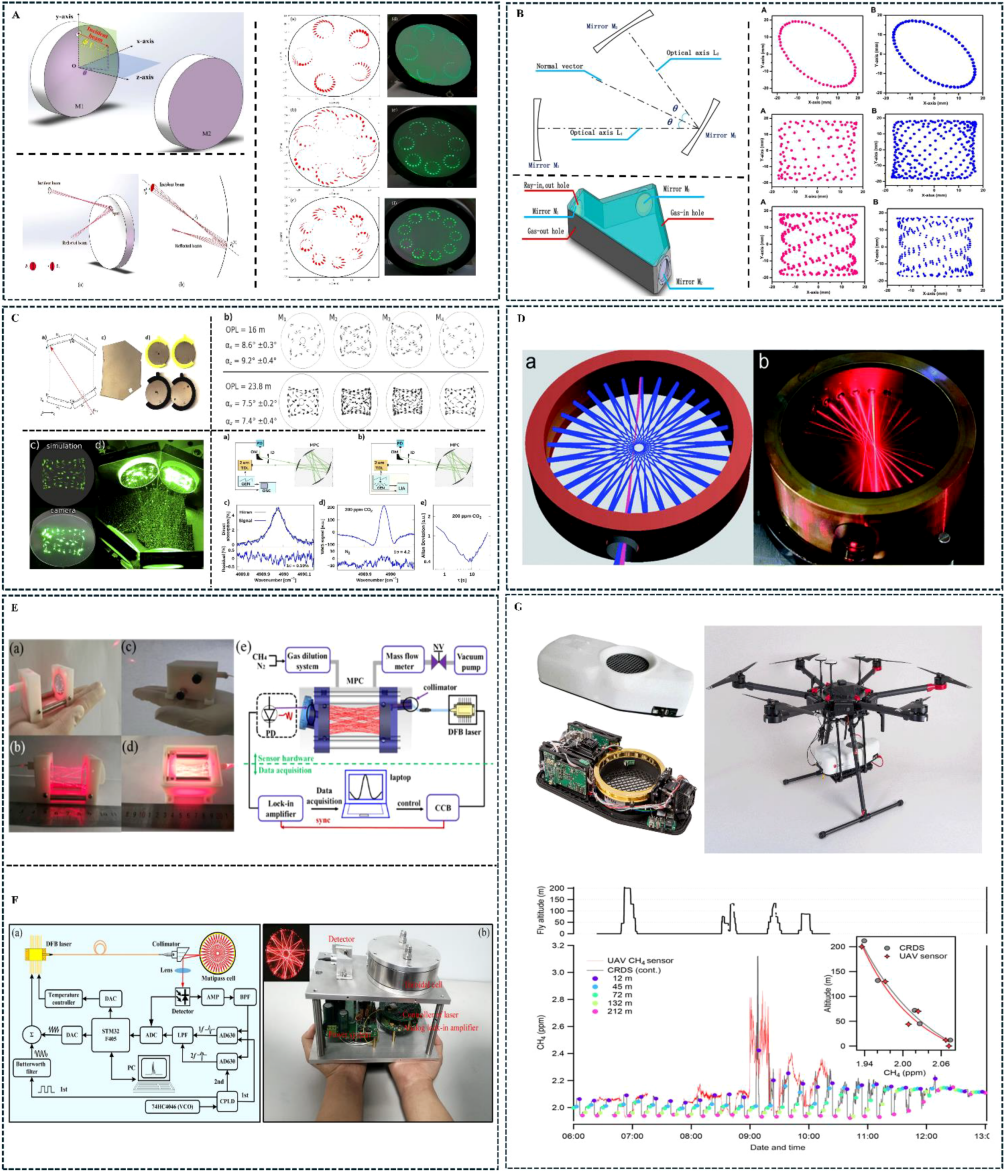
Application examples of finite-volume optical multi-pass cells. **(A)** (Liu et al., 2021); **(B)** (Cheng et al., 2022); **(C)** (Hudzikowski et al., 2021); **(D)** (Jouy et al., 2014); **(E)** (Cui et al., 2020); **(F)** (Feng et al., 2021); **(G)** (Tuzson et al., 2020).

The design theory for classic optical multi-pass cells dates to 1942, when John U. White at Esso Research Laboratory in the United States designed the White optical multi-pass cell using three spherical mirrors with the same curvature radius (White, 1942). In 1962, Herriott at Bell Labs developed the Herriott optical multi-pass cell with a ring-shaped spot reflection structure using two identical concave mirrors. In recent years, researchers have optimized the design of optical multi-pass cells by improving the type and number of mirrors, effectively increasing the utilization of mirrors. This has promoted the development of long optical path multi-pass cells in finite volumes, further advancing the design theory for finite volume long optical path multi-pass cells (Herriott et al., 1964). The reflection path of a laser between two spherical mirrors is typically described using the ABCD matrix and vector reflection principles. However, in optical multi-pass cells with dense spot patterns, the laser reflected between the two spherical mirrors is often off-axis, violating the paraxial conditions, making the ABCD matrix no longer applicable. Although the modified ABCD matrix can eliminate errors arising from the paraxial approximation, the ray tracing process itself involves repeated complex matrix calculations and iterations, making the design process very complicated. The literature has reported the application of relevant algorithms to improve the traditional optical multi-pass cell design theory. Liu et al. (2021) first used the Monte Carlo algorithm to determine the distance between two mirrors. Then, they used the Nelder-Mead simplex algorithm to locally optimize the incident parameters, performed ray tracing, and simulated the size and shape of the spots. Finally, they applied a clustering method to automatically select independent circular patterns, resulting in optimal circular patterns with five, seven, and nine circles. The numerical simulation results are shown in **Figure 5A**. In another study, Chen et al. (2022) proposed an intelligent method involving a parallel multi-population genetic algorithm to optimize the optical path length (OPL) and size of the traditional Herriott optical multi-pass cell. Following several iterations, they designed an optimal multi-pass cell with an OPL of 6.3 m, with mirror diameters and distances of 25.4 mm and 30.5 mm, respectively (Ozharar and Sennaroglu, 2017). introduced a new method using rotational symmetry, spherical aberration and offset-related focal lengths to create a unique optical design. This innovative design uses astigmatic mirrors to form spot patterns beyond the standard Lissajous figure.

**Figure 6:**
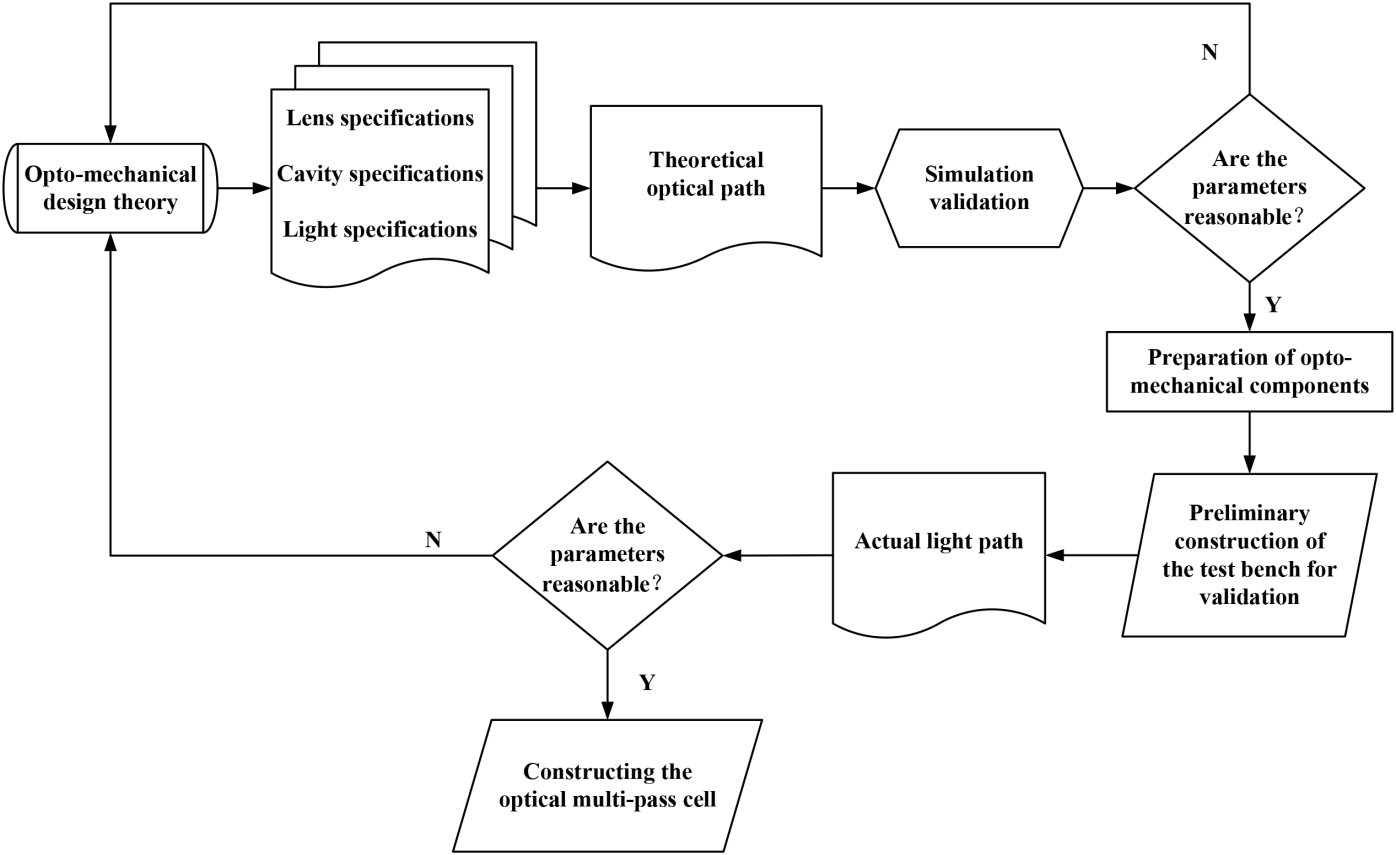
General optical multi-pass cell design process.

The practical development process of optical multi-pass cells typically includes theoretical design, parameter optimization, simulation verification, and practical validation, as shown in the design flowchart in **Figure 6**. In the practical design theory, optical multi-pass cells involve multiple variables, and the results vary under each parameter. The design typically follows the principle of optimal optical path design at the limit volume. The process begins with the determination of the volume and related dimensions based on engineering requirements. The ABCD matrix and its improved ray propagation theory are then used to calculate parameters such as the lens diameter and the spatial angular range of incident and outgoing light. The mirror distance is also dynamically adjusted. Simulations are then performed to calculate the optimal base length, with multiple iterations resulting in the best theoretical parameters and achieving the maximum optical path at the limit volume. Once the optimal theoretical parameters have been determined, multiple simulation verification iterations are carried out using well-established optical design software such as Zemax and Tracepro (Prabhat et al., 2023). If the simulation verification is successful, the optical-mechanical components are prepared and a test platform is built for optical experiments, with the aim of achieving the actual optical path. During gas detection experiments, researchers observed that the gas exchange rate is also a key indicator of the performance of optical multi-pass cells. This rate is primarily influenced by factors such as the structure of the gas chamber, the diffusion rate of the gas, and the speed of system signal processing. A higher gas exchange rate allows more substances to be fully interacted with by the light, resulting in more comprehensive detection of the sample gases. To avoid gas trapping and repeated laser detection caused by turbulence and eddy currents when gases pass through the optical multi-pass cell, researchers typically use fluid simulation software such as ANSYS and COMSOL. These programmers are used to simulate and validate the velocity of gas flow and the positions of pores (Liu, 2019; Yao et al., 2007).

In the field of optical path structure innovation, Wang et al. (2023) innovated the Herriott optical multi-pass cell with two 3mm-diameter holes at 90° in the second mirror. The outgoing light is reflected by M1 and M2 and returns to the cell to form a double-ring pattern with outer and inner circles. This is reflected 111 times, achieving an optical path of 73.926m. For multiple mirror structures and the White improved optical multi-pass cell. Cheng et al. (2022) proposed an improved ABCD matrix model. Using three non-coaxial mirrors, they developed a V-shaped multi-pass optical cell. This structure is very similar to a folded cavity in a laser cavity, meaning that the beam propagation matrix can be used for simulation and design. Ultimately, this achieves three effective OPLs of 49.6, 97.6 and 173.6 m and the spot patterns presented on the spherical mirrors M1, M2, and M3 of the three different multi-pass cells (MPCs) are shown in **Figure 5B**. Hudzikowski et al. (2021) used a genetic algorithm and four spherical mirrors to develop optical multi-pass cells with 16m and 23.8m optical path lengths. The MPC with an OPL of 23.8m was sealed and filled with 200ppm CO_2_ in nitrogen at 533 hPa. CO_2_ was detected at 2004nm using WMS, indicating a detection limit of 0.4ppmv in 10s. The spot pattern model for the best circular pattern presented by the four mirrors is shown in **Figure 5C**. The research and development of a new ring-type multi-pass cell was reported. Jouy et al. (2014) reported the development of a new ring-type multi-pass cell, using an 8cm diameter metal cylinder. To avoid optical fringes caused by stray light and main beam interference, they developed an absorption mask. The cylindrical chamber, with a volume of just 40 cm3, has an inner surface engraved with a ring mirror. Light enters the gas chamber through a small-angle incident hole directed toward the optical axis. After passing through multiple reflective holes, the light reflects up to 51 times, achieving a path length of 4m. The performance was evaluated by measuring the isotope ratio of CO_2_ under environmental abundance, with experimental results indicating a measurement accuracy of 2ppm over an integration time of 600s. The micro-sensor consists of a QCL mounted in an HHL, an annular multi-pass cell, and a QCD system with a preamplifier, as shown in **Figure 5D**.

The development of ultra-compact, small-volume optical multi-pass cells has also been reported. Cui et al. (2020) reported the development of an ultra-compact optical multi-pass cell device based on tunable diode laser absorption spectroscopy technology, using a 1.65µm near-infrared distributed feedback laser and a quartz tuning fork (QTF) as a thermal detector. The palm-sized methane (CH_4_) optical multi-pass cell device, with dimensions of just 78mm × 40mm × 40nm, achieved a minimum detection limit of 52 ppb within a 300ms integration time. The system structure is shown in **Figure 5E**. Feng et al. (2021) reported a compact ring-shaped optical multi-pass cell with an effective optical path length of 8.35m and 84 reflections, displaying a two-layer polygonal star-point pattern. Using a 1.653µm DFB laser, it detected methane in the atmosphere, achieving a detection limit of 22 ppb with a detection precision within a 57.8s integration time. The system is based on tunable diode laser absorption spectroscopy and a new dual-layer annular multi-pass cell, as shown in **Figure 5F**. Tuzson et al. (2020) developed a drone-mounted methane sensor. It uses a ring-shaped optical cell and a low-noise, intermittent continuous wave laser. Under stable laboratory conditions, the sensor achieved a detection limit of 0.1 ppb in 100 seconds. The CH_4_ sensor installed on the six-axis aircraft is shown in **Figure 5G**. Based on the above research, it is evident that optical multi-pass cell designs have more possibilities and flexibility. By increasing the number of mirrors or changing the types of mirrors, more optical multi-pass cell structures with different reflection types can indeed be obtained. However, the difficulty of obtaining design parameters increases significantly due to the influence of multiple parameters on the beam’s reflection behavior. Additionally, in ray tracing, the vector reflection principle offers greater advantages, as it eliminates the need to adjust the beam propagation matrix based on the placement of mirrors. The introduction of intelligent algorithms further enhances the accuracy and convenience of optimizing optical multi-pass cell parameter design.

### 4.3 High-precision detectors

In trace gas infrared sensing systems, the function of the detector is to capture the weak light intensity signal after infrared light has passed through the optical multi-pass cell and fully interacted with the target gas. This light signal is then converted into an electrical signal through the photoelectric effect. Due to the weak nature of the original electrical signal, it usually needs to be filtered and amplified by a signal amplifier before being input into an analog-to-digital(A/D) converter for signal acquisition and digitization. In the whole sensing system, the light intensity change signal captured by the detector is usually related to the concentration of the target gas. As such, the detector is a critical component for high-sensitivity gas detection, and its detection accuracy and response sensitivity directly influence the system’s performance. The most used infrared detectors for gas detection today include thermopile detectors, pyroelectric detectors, and various improved detectors based on new materials and structures. Pyroelectric detectors respond to changes in the crystal’s temperature when infrared radiation hits it. This generates a measurable charge signal which is then converted into a current signal. Thermopile detectors consist of stacks of thermocouple units. Materials with different thermoelectric properties and polarities form the cold and hot ends. A temperature difference generates charge carrier movement, which in turn produces a voltage output. Advances in microelectromechanical systems technology are enabling the development of smaller, more integrated detectors.

**Figure 7:**
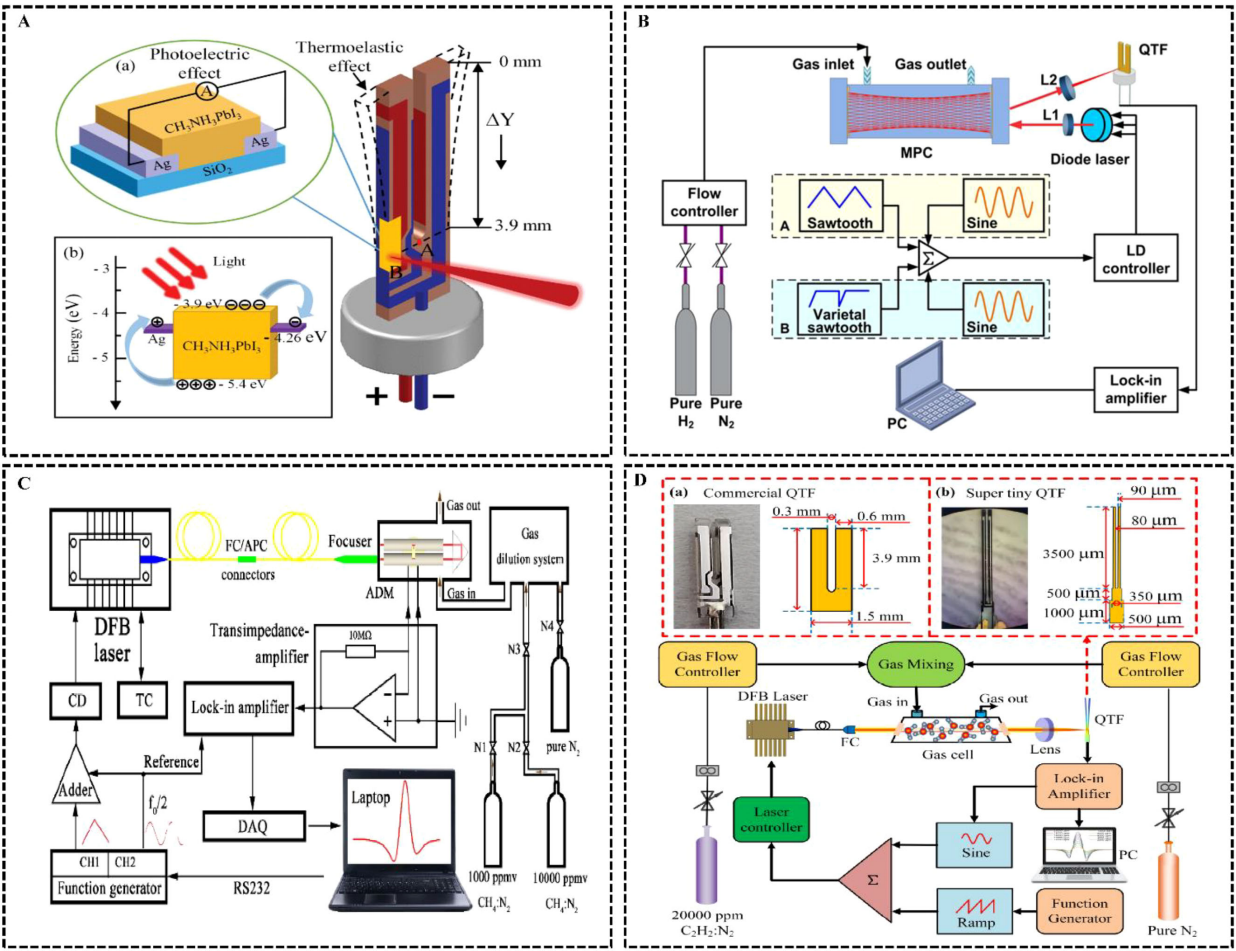
Application examples of high-precision detectors. **(A)** (Dai et al., 2023); **(B)** (Ma et al., 2023); **(C)** (Hu et al., 2019); **(D)** (Qiao et al., 2023).

In the field of infrared absorption spectroscopy trace gas sensors, some researchers have applied photoacoustic/photothermal technologies to enhance detectors and develop high-precision detectors. **Figure 7A** shows the structure of the opto-thermal-elastic coupling QTF sensor proposed by (Dai et al., 2023). This sensor utilizes a CH_3_NH_3_PbI_3_ perovskite. Perovskite thin film coated on the surface of a QTF and forms a Schottky junction between the QTF surface and a silver electrode. This structure exploits the optoelectronic and thermoelastic effects coupling between CH_3_NH_3_PbI_3_ and QTF, thereby enhancing the sensor’s detection sensitivity. Ma et al. (2023) developed a highly sensitive H_2_ detection system by combining infrared absorption spectroscopy with photoacoustic/photothermal technologies to detect H_2_ with weak absorption characteristics, achieving a detection limit of 45 ppm. **Figure 7B** illustrates the schematic of the H_2_ sensor platform based on photoinduced thermomechanical spectroscopy. Hu et al. (2019) addressed the issue of high acoustic coupling strength and detection sensitivity in quartz-enhanced photoacoustic spectroscopy by optimizing the structural parameters of the acoustic micro-resonator in the embedded off-beam quartz-enhanced photoacoustic spectroscopy spectrometer, based on principles of solid-state heat transfer and mechanics. This optimization led to an improved signal-to-noise ratio gain. Compared to the configuration using a bare QTF, dual-channel detection was achieved by embedding two resonant tubes into the QTF, resulting in approximately 40 times enhancement in the photoacoustic signal. The schematic of the embedded off-beam quartz-enhanced photoacoustic spectroscopy spectrometer with one or two resonant tubes is shown in **Figure 7C**. Qiao et al. (2023) developed a sensitive light-induced thermomechanical spectroscopy trace gas sensor using an ultra-miniature QTF with a prong length of 3500 µm, prong width of 90 µm, and a resonant frequency of 6.5 kHz, designed for detecting acetylene (C_2_H_2_) gas. Compared to commercial QTFs, the signal improved by 1.64 times, achieving a minimum detection limit of 190 ppb with an integration time of 220s. The schematic diagram of the ultra-miniature QTF is shown in **Figure 7D**. Wu et al. (2022) developed an 8mm long clamped quartz tuning fork-enhanced photoacoustic spectroscopy system, which enhances the acoustic wave coupling efficiency by introducing a wavefront-shaped aperture with a diameter up to 1mm, while keeping the Q factor > 104, increasing the laser beam focusing area by more than ten times compared to the standard QTF, and achieving approximately 30 times signal-to-noise enhancement compared to the dual-tube micro-resonator in traditional Quartz enhanced photoacoustic spectroscopy systems. Wang et al. (2025) designed a quadrupole quartz tuning fork with more prongs to increase stress concentration for better acoustic detection. This also generated stronger signals, with a minimum detection limit of 96 ppb. Simon et al. (2025) coherent control technology was introduced in quartz-enhanced photoacoustic spectroscopy. The main approach was to adjust the laser pulse timing to match half the oscillation period of the tuning fork while keeping the laser output frequency constant. This adjustment ensures that the laser pulse reaches the gas molecules between the tuning fork arms, causing the gas to heat and expand, producing a reverse force on the tuning fork’s motion. A proof-of-concept platform was successfully established for analyzing methane gas using a quartz-enhanced photoacoustic spectroscopy chamber, showing that the coherent control method keeps spectral fingerprints clear and stable.

## 5 Application of infrared absorption spectroscopy trace gas sensors for crop pest detection in agriculture

When developing and designing infrared absorption spectroscopy trace gas sensors for pest detection, the first step is to collect the characteristic VOCs “fingerprint” gases emitted by plants under pest stress. Once the characteristic fingerprint gases have been identified, their absorption spectra are simulated under normal pressure and temperature conditions using the HITRAN database. The light source is chosen based on its absorption spectra, ensuring strong absorption features within its wavelength tuning range. Subsequently, a high-precision discrete optical multi-pass cell was designed and constructed, integrating optical and mechanical design principles. This cell was engineered to maximize the number of cycles and reflections of the light emitted by the source, thereby ensuring full interaction with the target gas within the confined volume of the gas cell. During gas sampling, techniques such as headspace, diffusion, bubblers, or pre-concentrators are used to introduce the gas into the optical multi-pass cell, with the gas pump controlling the sensor response during specific cycles. The laser’s light passes through the gas in the optical multi-pass cell, where it is absorbed and detected. The gas type and concentration are determined by analyzing the signal data. This process also involves sensor calibration using known concentrations of target gases to analyze detection accuracy and performance. Following calibration, gas absorption is derived using baseline fitting in conjunction with the Lambert–Beer law to determine the concentration of the gas under test from its absorbance.

### 5.1 Application of infrared absorption spectroscopy trace gas sensors

**Figure 8:**
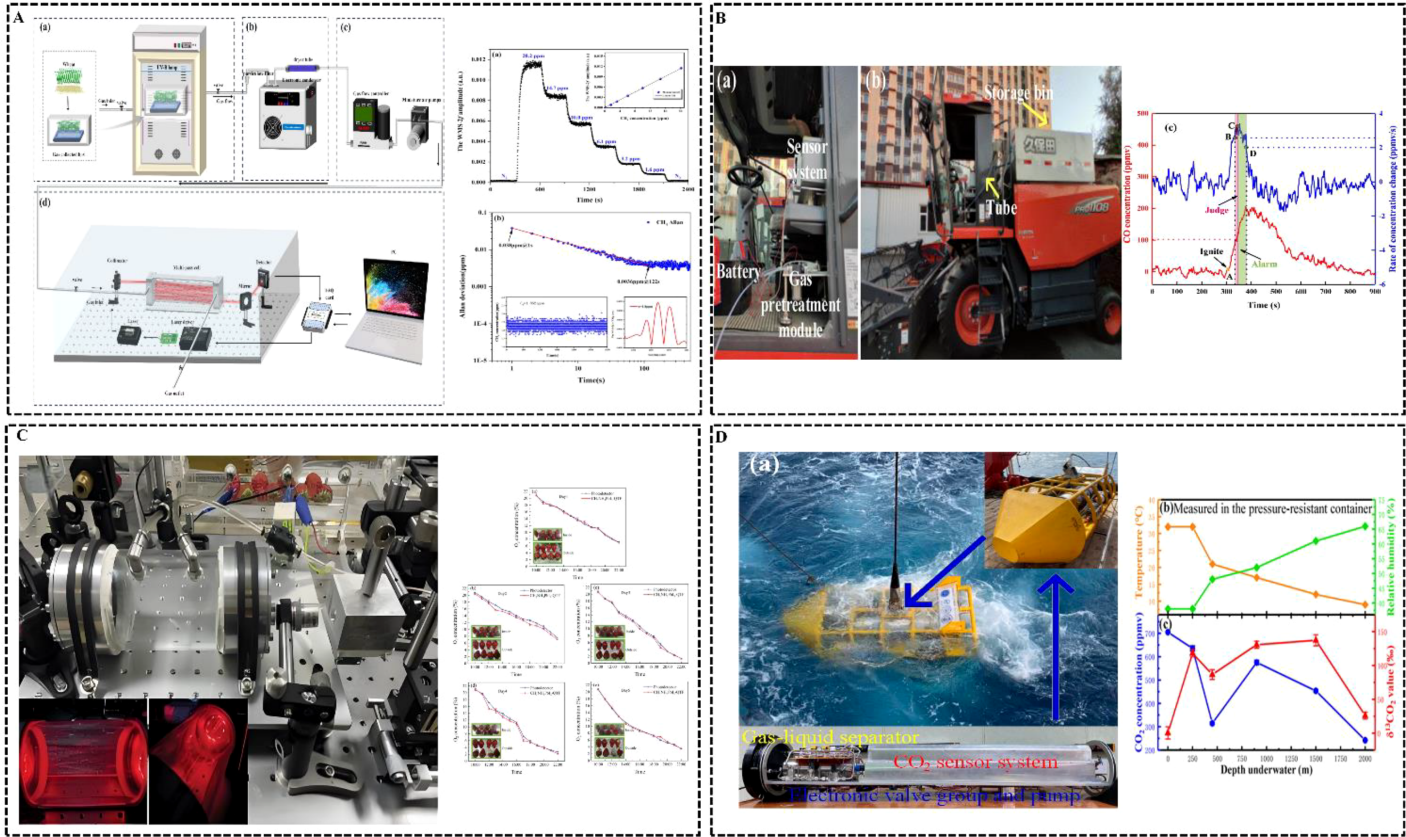
Application examples of infrared absorption spectroscopy trace gas sensors in agriculture. **(A)** (Zhang et al., 2025); **(B)** (Li et al., 2023); **(C)** (Dai et al., 2023); **(D)** (Liu et al., 2020).

Several trace gas sensors using infrared absorption spectroscopy have been reported in the literature. These sensors can monitor agricultural production and detect specific gases in marine environments. For instance, the presence of endogenous methane gas is a key indicator of whether wheat experiences high-temperature and UV stress during the tillering stage. Zhang et al. (2025) developed an early recognition and prediction model by combining hyperspectral technology with infrared absorption spectroscopy to predict endogenous methane gas produced by wheat under heat stress (HS) and ultraviolet-B stress, achieving an accuracy of 98.88%. The structure of the detection system is shown in **Figure 8A**. Li et al. (2023) developed a mid-infrared CO sensor for fire warnings in cotton harvesting. The Lod was 0.83 ppm, and the system worked well at -40 to 85 °C. The mid-infrared CO and CO_2_ dual-gas sensor system for cotton harvesters is shown in **Figure 8B**. Dai et al. (2023) used infrared absorption spectroscopy and LITES technology to monitor changes in O_2_ concentration and assess strawberry spoilage during storage. The strawberry gas detection system is shown in **Figure 8C**. Liu et al. (2020) developed a system for measuring dissolved CO_2_ in seawater using a rare CO_2_ sensor based on tunable laser absorption spectroscopy. This system used a 4319 nm laser and an optical multi-pass cell and was integrated with a gas-liquid separator. It was deployed in the South China Sea at 30 Torr, validating the sensor’s ability to analyze dissolved gases *in situ*. The deep-sea dissolved CO_2_ detection system is shown in **Figure 8D**.

### 5.2 Pest species identification methods

Chemometrics has shown unique application value in several fields, including tea flavor identification, food adulteration detection, and origin tracing (Fang et al., 2019; Wang et al., 2022). Pest species identification relies on gas molecular fingerprints as key discriminative information. Chemometrics, as an important tool linking infrared absorption spectroscopy signals with target attributes, plays a critical role in data-driven modelling. Following the sensitive detection of pest-induced VOCs by infrared spectroscopy, chemometrics is employed to extract spectral features, build discrimination models, and ultimately match the spectral patterns to specific pest species through statistical analysis. The basic process consists of data pre-processing, feature extraction, modelling analysis, and model validation. Common data preprocessing methods include standard normal variation transformation, multiplicative scatter correction, and first- and second-order derivative transformations, which help reduce spectral noise, correct baseline drift, and improve data quality and stability. During feature extraction, principal component analysis and linear discriminant analysis are extensively used for spectral dimensionality reduction and sample classification tasks (Monika et al., 2024). Partial least squares regression is often used to establish quantitative relationships between spectral variables and target attributes, especially for handling high-dimensional and multicollinear data. To meet the demand for nonlinear feature modelling, methods like artificial neural networks and support vector machines have been introduced in spectral analysis in recent years, significantly enhancing the model’s ability to express complex systems and improve generalization performance.

Wang et al. (2020) studied how to detect wood-boring pests hidden inside tree trunks. They analyzed pest sample gases using GC-MS and identified four key gases as molecular fingerprints for the MIP molecular template. They then developed a statistical model using data from the QCM sensor array to differentiate the gases emitted by cedar trunks infected by the pests, and uninfected trunks. PCA and LDA were used to reduce dimensions and visualize the data. K-nearest neighbors, probabilistic neural networks, and support vector machines were used to establish pattern recognition models. The results confirmed that chemometric pattern recognition methods, combined with the QCM sensor array, could effectively identify the infestation of wood-boring insects, such as the striped pine woodborer and cedar bark beetle, in cedar trunks. Cardoso et al. (2025) used chemometric methods with NIR, MIR spectroscopy and RP-HPLC to analyze 56 traditional Mina’s cheese samples from different regions in Minas Gerais, Brazil. They obtained fingerprint spectra for cheeses from different regions and successfully traced their origin. PCA was used to assess similarities and differences in WSP extracted from cheeses produced in different regions. PLS-DA was used to establish a model that classifies different regions using NIR and MIR spectral data. Thus, chemometric methods provide efficient and sensitive tools for identifying plant pest damage, supporting precision agriculture.

### 5.3 Pest infestation scale determination methods

Multivariate statistical analysis plays a crucial role in pest scale determination methods. Plants under pest stress release specific VOCs, and temporal variations in the concentrations of these gases can be significant indicators when evaluating pest severity and trends in spatial spread (Ricardo et al., 2019). VOCs released by plants are typically collected over continuous time periods at fixed intervals. The concentration of target gases (such as alcohols, esters, terpenes, etc.) is dynamically monitored to analyze their variations over time. Based on the response amplitude of VOCs concentrations, release rates, and peak times, multivariate statistical methods such as cluster analysis, discriminant analysis, and principal component regression can be employed to create pest severity classification models (e.g., mild, moderate, severe). These maps can predict the spatial scale of infestations, enabling regional monitoring and providing a quantitative basis for precision pest control decisions.

Henderson et al. (2010) used a portable electronic nose to detect stink bugs in cotton. Their study found a strong correlation between pest density and sensor response. Zhou et al. (2022) used infrared spectroscopy to study grape storage spoilage detection by monitoring ethanol gas concentrations. Their research found that as storage time increased, the ethanol absorption peak intensity grew. PCA analysis revealed distinct clustering at different storage times, indicating significant changes in ethanol concentration over time. The qualitative model developed with PLS-DA accurately classified grape samples into “fresh,” “slightly spoiled,” or “severely spoiled,” achieving 100.00% accuracy. Schaub et al. (2010) used proton transfer reaction mass spectrometry and gas chromatography-mass spectrometry to monitor the release characteristics of VOCs from poplar trees after feeding by fall cankerworms in a field environment. Their results showed that VOCs concentrations and compositions were closely related to pest density. Cai et al. (2015) used solid-phase microextraction combined with GC-MS technology to analyze the dynamic changes in VOCs during aphid infestation in soybeans. They found that certain key compounds (such as green leaf volatiles and terpenes) increased significantly as the pest damage intensified, indicating that VOCs concentrations can be used as an indicator of pest intensity. Milton et al. (2022) further systematically compared VOCs emission profiles from different crop varieties under similar pest infestations. They suggested that changes in VOCs components could be used both for screening variety resistance and for developing pest severity recognition models. Collectively, these studies demonstrate that continuous monitoring of VOC dynamics, combined with statistical modelling, enables not only qualitative pest identification but also quantitative classification of pest intensity and spatial prediction. This provides a theoretical foundation and methodological framework for regional pest monitoring and precision control.

## 6 Challenges and the perspectives

Sensors as a crucial component of future agricultural production systems, are necessary for accurately assessing crop conditions. As a key infrastructure for sensing and monitoring environmental information in agricultural fields, gas detection sensors for pests provide essential data for agricultural management. Although significant progress has been made in researching infrared absorption spectroscopy trace gas sensors, the complex growth environment of this technology still presents major challenges. In practical applications, these sensors encounter numerous bottlenecks and obstacles when it comes to detecting crop pests. Further research is required to promote the application of infrared absorption spectroscopy gas sensor technology in crop pest monitoring. The following sections will discuss this in detail.

(1) Following pest infestation, plants release biogenic VOCs that are multi-component, low-concentration, strongly collinear, and have highly overlapping spectral bands; high-resolution infrared spectroscopy can resolve fine spectral structures in the fingerprint region (approx. 600–1500 cm^-^¹) and functional group region (approx. 2800–3200 cm^-^¹), providing high-dimensional and continuous spectral information for gas mixtures. However, spectral overlap and baseline drift significantly increase the difficulty of analysis, and the introduction of chemometrics can effectively solve the problems of spectral overlap and high-dimensional collinearity. Regarding feature compression and redundancy removal, methods such as PCA and ICA are usually adopted to project high-dimensional spectra into a low-dimensional feature space to highlight dominant variation patterns and suppress noise and background interference. For mixture decoupling and quantitative modeling problems, methods like PLSR and MCR-ALS are commonly used to achieve simultaneous quantification of multiple components under spectral overlap, separate weak feature gases from complex backgrounds, and establish a stable mapping between concentration changes and spectral responses. In terms of pattern recognition and fingerprint discrimination, LDA, SVM, Random Forest, or deep learning models are usually used, treating the overall spectral response of VOCs as a “gas fingerprint” to distinguish different physiological states, stress types, or pest stages. Despite the promising prospects of this technology, several challenges remain, including the sensitivity of spectral data to environmental temperature, humidity, and background gas fluctuations; the dependence of chemometric models on sample diversity and the size of calibration sets; and the insufficient robustness of laboratory models when transferred to on-site online monitoring applications. Consequently, future research should focus on continuously improving spectral stability, enhancing model generalization capabilities, and promoting the fusion and application of multi-source information.

(2) Beyond the prerequisites of miniaturization, lightweight design, and low power consumption, pest gas sensors must also exhibit superior platform compatibility. However, regarding platform compatibility, the sensor optical system is easily susceptible to interference, specifically manifested as micro-displacement or angular deviation of the light source, mirrors, and detectors, leading to effective optical path fluctuation and causing unstable absorption signals. When applied to fixed base station point monitoring, good environmental adaptability is needed, such as high-reflectivity and pollution-resistant optical mirror materials and high-airtightness gas path systems, while aerial platform integration faces the double impact of vibration coupling and airflow disturbance on the optical system and gas sampling process, existing vibration damping schemes mostly rely on rubber damping pads, elastic suspension, or passive vibration isolation structures, which have limited suppression ability for wide-band random vibrations and are difficult to balance between weight and structural stiffness, restricting the miniaturization and long-term stable operation of the system. Regarding airflow disturbance, the strong turbulence and periodic pulsating airflow generated by UAV rotors will significantly interfere with sensor air intake conditions; existing airflow regulation methods mostly adopt simple windshields, extended intake pipes, or flow stabilizing chamber structures, which can weaken airflow impact to a certain extent but are often accompanied by problems like extended response time, gas stagnation, and sample dilution, making it difficult to achieve sampling control that balances real-time performance and accuracy under dynamic flight conditions. Therefore, future research needs to continue to improve in aspects like lightweight active vibration damping or optical path self-calibration technology, directional sampling and pressure compensation strategies facing rotor environments, and developing adaptive spectral algorithms to correct measurement deviations induced by non-steady-state sampling conditions.

(3) To meet the requirements of various detection scenarios and to avoid interference from other gases, corresponding pre-sampling devices need to be developed for different parts of the crop. For example, in the case of underground pests, a gas pre-sampling probe suitable for the crop root zone should be developed. This probe should possess soil penetration capabilities and depth positioning functions, allowing for high-precision collection and analysis of metabolic gases from underground pests, enabling early warning and continuous monitoring (Tholl et al., 2021). A gas sensor network consists of multiple gas sensors distributed at different locations, enabling more comprehensive gas detection. Current research is focused on exploring the development of wireless power technologies and large-scale transmission networks to enable the networking and intelligence of monitoring systems. Compared to a single sensor, sensor group networks offer significant advantages in monitoring range, data accuracy, and system stability. The collected data must be transmitted to a cloud server to support complex data processing, big data mining, and remote monitoring, thus enabling efficient environmental sensing and management.

(4) Although single detection methods have some advantages in specific environments, their limitations gradually become apparent in the face of the complexity and variability of natural environments. To achieve more comprehensive and precise perception of crop health status, future research will focus on developing a collaborative monitoring model based on multi-sensor fusion (Gu et al., 2025).Multi-sensor fusion systems integrate technologies like gas detection, image recognition, and remote sensing monitoring to sense crop growth status from both macro and micro levels, improving response speed and prediction capabilities for pests, nutrient deficiencies, and environmental stress. By combining multi-sensor fusion technology with cloud platforms and big data systems, forward-looking management tools for agriculture will be provided, advancing smart agriculture from “perception” to “cognition” and “decision-making. Therefore, multi-sensor fusion monitoring systems are not only a key technology to enhance the intelligence level of agriculture but will also play a central role in future smart agriculture, helping to drive efficient, sustainable, and precise agricultural development.

## 7 Conclusion

This review systematically evaluates the progress of research on crop pest detection methods and equipment, discussing the application mechanism of volatile organic gases in monitoring plant pests, as well as key scientific issues such as the principle of infrared absorption spectroscopy gas detection and high-precision optical multipass cell design strategies. Overall, pest monitoring technology based on infrared spectroscopy is transitioning from laboratory verification to complex field environment application; its development key lies in revealing the biological mechanism of pest-induced gas release, establishing stable mixed gas spectral fingerprint discrimination methods, and breaking through technical bottlenecks such as on-site environment interference, non-steady-state sampling, and multi-platform integration. Future development trends mainly include improving detection sensitivity and selectivity, achieving miniaturization and portability, combining intelligent data processing for early warning, and integrating with various sensing means to support precision prevention and control. However, field application continues to face significant scientific and technological challenges:

(1) Biological discriminability of characteristic gas spectral fingerprints. It is urgently needed to clarify the physiological mechanism of pest feeding-induced VOC release, clarify its correspondence with pest species, population density, and stress stage, and solve the problem that pest signals are difficult to distinguish from spectral responses of other abiotic stresses.

(2) Stable recognition methods for mixed gas spectra in complex field environments. Aiming at field low-concentration, multi-component, and strong background interference conditions, it is necessary to develop mixed gas fingerprint analysis methods based on high-resolution infrared spectroscopy and chemometrics, breaking through the limits of spectral overlap and non-steady-state sampling on quantification and discrimination accuracy.

(3) System robustness and platform adaptability for field applications. In practical deployment, infrared spectral sensors must address stability issues arising from environmental fluctuations, airflow disturbances, and multi-platform integration to achieve miniaturization, low power consumption, and long-term online operation, ultimately providing reliable data support for early pest warning and precision control.
